# Highly efficient gene knockout by injection of TALEN mRNAs into oocytes and host transfer in *Xenopus laevis*

**DOI:** 10.1242/bio.201410009

**Published:** 2015-01-16

**Authors:** Keisuke Nakajima, Yoshio Yaoita

**Affiliations:** Division of Embryology and Genetics, Institute for Amphibian Biology, Graduate School of Science, Hiroshima University, Higashihiroshima 739-8526, Japan

**Keywords:** Host-transfer, TALEN, Genome editing, Targeted gene knockout, *Xenopus laevis*

## Abstract

Zinc-finger nucleases, transcription activator-like effector nucleases (TALENs) and the CRISPR/Cas (clustered regularly interspaced short palindromic repeats/CRISPR-associated proteins) system are potentially powerful tools for producing tailor-made knockout animals. However, their mutagenic activity is not high enough to induce mutations at all loci of a target gene throughout an entire tadpole. In this study, we present a highly efficient method for introducing gene modifications at almost all target sequences in randomly selected embryos. The gene modification activity of TALEN is enhanced by adopting the host-transfer technique. In our method, the efficiency is further improved by injecting TALEN mRNAs fused to the 3′UTR of the *Xenopus DEADSouth* gene into oocytes, which are then transferred into a host female frog, where they are ovulated and fertilized. The addition of the 3′UTR of the *DEADSouth* gene promotes mRNA translation in the oocytes and increases the expression of TALEN proteins to near-maximal levels three hours post fertilization (hpf). In contrast, TALEN mRNAs without this 3′UTR are translated infrequently in oocytes. Our data suggest that genomic DNA is more sensitive to TALEN proteins from fertilization to the midblastula (MBT) stage. Our method works by increasing the levels of TALEN proteins during the pre-MBT stages.

## INTRODUCTION

Targeted gene disruption using either transcription activator-like effector nucleases (TALENs) (Christian et al., 2010; Li et al., 2011) or the CRISPR/Cas (clustered regularly interspaced short palindromic repeats/CRISPR-associated proteins) system (Jinek et al., 2012) is a powerful method for determining the function of a specific gene. TALENs are composed of a nuclear localization signal, an N-terminal domain, a target DNA-binding domain, a C-terminal domain and the nuclease domain of FokI, and are nuclear proteins that bind to target DNA to form dimers in the nuclease domain, inducing double strand cleavage. In the CRISPR/Cas system, a single synthetic guide RNA that is partially complementary to the target sequence co-operates with the Cas9 protein to cut the target site. The double strand breaks in the target gene are frequently repaired by non-homologous end-joining, which is an error-prone repair process, leading to nucleotide deletion and/or insertion.

To elucidate gene function during development, especially during early embryogenesis, a highly efficient gene knockout method is necessary to modify all of the loci of a target gene in randomly selected F0 embryos because of the chimeric nature of the embryos and technical difficulties associated with the genotyping of tiny embryos (Nakajima et al., 2012). It should be possible to efficiently increase the level of an effector protein during early embryogenesis. In *Xenopus laevis*, the host-transfer technique can be used to inhibit maternal gene function. This technique is performed by injecting antisense oligonucleotides into the oocytes, transferring the oocytes into an egg-laying host female, and fertilizing them *in vitro* (Holwill et al., 1987). If TALEN mRNAs are injected into oocytes instead of antisense oligonucleotide, the translated proteins can be expressed at high levels during early embryogenesis, enhancing their mutagenic activity.

In this study, we establish a highly efficient gene knockout method using oocyte injection of TALEN mRNAs fused to the 3′ untranslated region (3′UTR) of the *DEADSouth* gene in conjunction with the host-transfer technique. Almost all of the examined target DNA sequences in randomly selected embryos were modified by this procedure.

## MATERIALS AND METHODS

### Animals and eggs

Wild-type *X. laevis* were maintained at 20°C. Eggs were manually removed from the adult females by squeezing, a half day after the injection of 450 U of human chorionic gonadotropin (ASKA, Tokyo, Japan). A testis was dissected from a male and suspended in 1 ml of 1 × MBS (88 mM NaCl/1 mM KCl/1 mM MgSO_4_/5 mM HEPES (pH 7.8)/2.5 mM NaHCO_3_) containing 0.1% bovine serum albumin. The testis suspension (100–300 µl) was placed on the eggs, mixed, and allowed to settle in 0.1 × MMR [100 mM NaCl/2 mM KCl/2 mM CaCl_2_/1 mM MgCl_2_/5 mM HEPES (pH 7.4)] at 22°C for 8 min. The *in vitro* fertilized eggs were then de-jellied in 3.5% cysteine and washed three times in 0.1 × MMR. The fertilized eggs were maintained in 0.5 × MMR containing 6% Ficoll PM400 (SIGMA) at 22°C until microinjection. Embryos were staged according to the Nieuwkoop and Faber (Nieuwkoop and Faber, 1956). All animals were maintained and used in accordance with the guidelines established by Hiroshima University for the care and use of experimental animals.

### Oocyte collection and host-transfer technique

Oocyte collection and host transfer were performed as previously described (Holwill et al., 1987), with minor modifications. Briefly, female frogs that had not been stimulated to lay eggs in the past six weeks were used for oocyte collection. Their ovaries were removed and placed in 0.1 × MBS. The defolliculated oocytes were cultured at 18°C in 67% modified L-15 medium (ICN Biomedicals Inc.) with 0.04% BSA, 100 U/ml penicillin G sodium salt (Sigma), 100 µg/ml streptomycin sulfate (Sigma), and 10 mM HEPES (pH 7.1). Maturation was initiated by adding progesterone to the culture medium to a final concentration of 2 µM and culturing at 16°C for 16 hours. Cultured oocytes were stained with vital dye(s) for 15 min before washing in the culture medium. The following dyes were used and were dissolved in culture medium; green: Nile Blue A (SIGMA) 0.001% mixed with Bismarck Brown (Wako) 0.01%, brown: Bismarck Brown 0.01%, Mauve: Nile Blue A 0.001% mixed with Neutral Red (SIGMA) 0.0025%, red: Neutral Red 0.0025%. Transfer of the oocytes into the host female was performed as previously described (Olson et al., 2012).

### Construction of the TALENs

A DNA fragment containing *mCherry* was amplified from the pmCherry-N1 vector (Clontech) using *TaKaRa EX Taq* Hot Start Version (TaKaRa) and the primers TALENmCherry5′ (5′-GACGGGTGCCCCCCTGGAGACGGGCGTCTCCAACGTGAGCAAGGGCGAGGAGGA-3′) and TALENmCherry3′ (5′-TGACTAGTTGGGATCCCTTGTACAGCTCGTCCATGCC-3′) with a three-step protocol [(95°C, 20 s; 55°C, 30 s; 72°C, 60 s) × 23; 72°C, 15 min). The DNA fragment was inserted into the Esp3I and BamHI sites of the TALEN vectors with obligate heterodimeric *Fok*I (ELD/KKR) (Lei et al., 2012) using the In-Fusion Advantage PCR Cloning kit (Clontech) to obtain pTALEN-mCherry-ELD and pTALEN-mCherry-KKR.

A DNA fragment containing the *X. tropicalis DEADSouth* 3′UTR was amplified using *TaKaRa LA Taq* Hot Start Version (TaKaRa) and the primers XhoXtDS5 (5′-GGCTCGAGTAGGTGTGGCAGCACAA-3′) and XtDS3BamHI (5′-GGGGATCCGAATTTCCCTAAATTGTCTTTACAAAG-3′) with a three-step protocol [94°C, 60 s; (98°C, 10 s; 55°C, 30 s; 72°C, 30 s) × 35; 72°C, 10 min]. The product was digested with XhoI and BamHI and ligated into pEGFP-C3 (Clontech). This *DEADSouth* 3′UTR fragment was inserted into the XbaI site of pTALEN-ELD and pTALEN-KKR using the In-Fusion Advantage PCR cloning kit to obtain pTALEN-ELD-DS and pTALEN-KKR-DS, respectively.

The DNA binding domains were designed to target the first exon of the *X. laevis tyrosinase* gene. The TALEN binding sites (left: 5′-GACTTTGCCCATGAAGCTCCA-3′ and right: 5′-GCAGCAAGAAGTACCGGTG-3′) were located in the conserved region between the DNA sequences of *X. laevis tyrosinase* homoeologs, the accession numbers AY341764 and BI442159. The TALEN repeats were assembled as previously described (Cermak et al., 2011) with minor modifications (Nakajima et al., 2013) and inserted into pTALEN-ELD, pTALEN-KKR, pTALEN-ELD-DS, pTALEN-KKR-DS, pTALEN-mCherry-ELD and pTALEN-mCherry-KKR to generate Tyr-TALEN, Tyr-TALEN-DS and Tyr-TALEN-mCherry expression constructs. The *DEADSouth* 3′UTR fragment was inserted into the XbaI site of the Tyr-TALEN-mCherry expression constructs using the In-Fusion Advantage PCR Cloning kit to obtain Tyr-TALEN-mCherry-DS expression constructs as described above.

A DNA fragment containing the *X. laevis type-5 actin* 3′UTR was amplified using *TaKaRa LA Taq* Hot Start Version (TaKaRa) and the primers actin3UTR5 (5′-GGGTCTAGACTGCTAGCAGATGCGT-3′) and actin3UTR3 (5′-GGGGCGGCCGCAGCGCTTTATTTTATCCTTACAG-3′) with a three-step protocol [94°C, 60 s; (98°C, 10 s; 55°C, 30 s; 72°C, 30 s) × 35; 72°C, 10 min]. The product was purified and digested with XbaI and NotI, and ligated into the XbaI and NotI sites of Tyr-TALEN-mCherry expression constructs to obtain Tyr-TALEN-mCherry-actin constructs.

### RNA microinjection

The mRNAs were transcribed *in vitro* from the NotI-digested Tyr-TALEN and Tyr-TALEN-mCherry expression constructs and from the XbaI-digested Tyr-TALEN-DS and Tyr-TALEN-mCherry-DS expression constructs using the mMESSAGE mMACHINE SP6 kit (Ambion), and were dissolved in Nuclease-Free Water (Ambion). Oocytes or fertilized eggs were injected with 10 nl of 25 ng/µl each of Tyr-TALEN (or Tyr-TALEN-DS) mRNAs and 25 ng/µl mCherry mRNA. Alternatively, they were injected with 10 nl of 25 ng/µl each of Tyr-TALEN-mCherry (or Tyr-TALEN-mCherry-DS) mRNAs. The embryos were raised at 20°C in 0.5 × MMR.

### DNA purification

A group of oocytes was homogenized in 18 µl of 50 mM NaOH per oocyte and incubated for 10 min at 100°C. The homogenate was neutralized using 2 µl of 1 M Tris-Cl (pH 8.0) per oocyte and centrifuged at 15,000 × g for 10 min at 4°C. The supernatant was mixed with phenol and chloroform vigorously, and centrifuged. The aqueous phase was transferred into a new tube and added to 30 µl of Direct Purification Buffer and 25 µl of Wizard PCR Preps DNA purification resin (Promega) per oocyte. The DNA was purified according to the manufacturer's instructions. Mutation analysis was performed using the entire DNA solution prepared from the group of oocytes.

Each randomly selected stage 4–8 embryo was frozen at −20°C for more than 30 min and thawed. This procedure was repeated, and the genomic DNA was extracted from each embryo using the SimplePrep reagent for DNA (TaKaRa) using the procedure of the manufacturer. The mutation analysis was performed using 75%, 33%, and 6.7% of the genomic DNA solution prepared from the individual stage 4, stage 6, and stage 7.5–8 embryos, respectively.

Each of the randomly selected stage 13–47 tadpoles was homogenized in 90 µl of 50 mM NaOH and incubated for 10 min at 95°C. The homogenate was neutralized with 10 µl of 1 M Tris-Cl (pH 8.0) and centrifuged at 1500 × g for 10 min at room temperature. The supernatant was mixed with phenol and chloroform vigorously, and centrifuged. The mutation analysis was conducted using 1% of aqueous phase prepared from each tadpole.

### Mutation analysis

A DNA fragment containing the target site was amplified using the EmeraldAmp MAX PCR Master Mix (TaKaRa) and the primers Tyr-F (5′-GTCATAGTCACTGGGACCTAC-3′) and Tyr-R (5′-TCATCTGTGCAAATGTCACAACC-3′) with a three-step protocol [(95°C, 30 s; 58°C, 30 s; 72°C, 30 s) × 20–40). The second round of PCR was performed using the primers Tyr-F2 (5′-CGTGTATGACCTCTTTGTGTGG-3′) and Tyr-R2 (5′-CTGTGCAAATGTCACAACCTTG-3′) with a three-step protocol [(95°C, 30 s; 58°C, 30 s; 72°C, 30 s) × 20–40]. The PCR products were subcloned into the pGEM-T Easy vector (Promega) and the nucleotide sequences were subsequently determined. All four primers are designed to the conserved region between *tyrosinase* homoeologs, AY341764 and BI442159. The amplification efficiencies during PCR using these primer sets were similar (supplementary material Fig. S4).

### Western blot analysis

Five oocytes, embryos or tadpoles were homogenized, and the total protein equivalent to one-tenth of an oocyte, embryo or tadpole was subjected to SDS-polyacrylamide gel electrophoresis. Each blot was separated into an upper part and a lower part, which were probed with a rabbit anti-DsRed polyclonal antibody (Clontech) diluted 1:1000 and a rabbit anti-actin antibody (Sigma-Aldrich) diluted 1:2000, respectively. A phosphatase-labeled goat anti-rabbit IgG antibody (KPL) diluted 1:10,000 was used as a secondary antibody. Reactive bands were visualized by treatment with CDP-Star Chemiluminescence Reagent (PerkinElmer).

## RESULTS

**Fig. 1.**
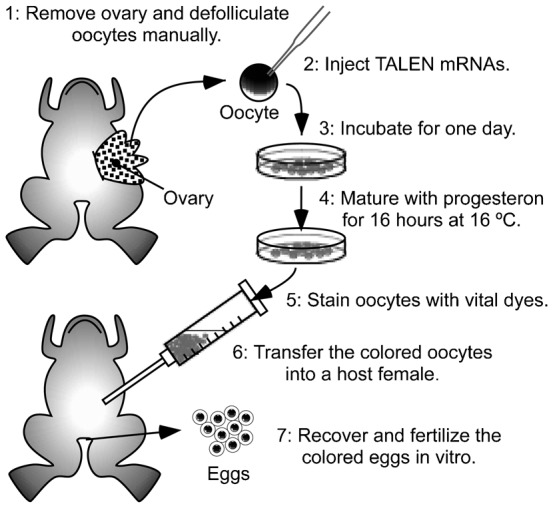
Schematic representation of the experimental procedure.

We reasoned that the efficiency of genomic editing would be improved if oocytes were injected with TALEN mRNAs, treated with progesterone for maturation, restored into a host female, and fertilized, as shown in Fig. 1. We expected that the injected TALEN mRNAs would be translated in the oocytes and that the TALEN proteins would digest the genomic DNA before and after fertilization. To test this, Tyr-TALEN constructs were designed to target the *X. laevis tyrosinase* gene using the TALEN scaffold with obligatory heterodimeric nuclease domains (Lei et al., 2012; Nakajima and Yaoita, 2013). Tyrosinase is indispensable for melanin synthesis. The disruption of all *tyrosinase* gene loci results in an albino phenotype, which can be easily observed (Ishibashi et al., 2012; Nakajima et al., 2012). Oocytes were injected with mRNAs transcribed from Tyr-TALEN constructs, transferred into a host female after maturation by progesterone, and fertilized. The embryos were sacrificed at various times for mutation analysis of the target DNA. In the CRISPR/Cas system, a single synthetic guide RNA is injected into embryos with *Cas9* mRNA, which binds to the target site and recruits the Cas9 protein to produce double-strand breaks. The DNA-targeting RNA and the translated protein from the mRNA work together in this system, and it is easy to monitor the stability of only the translated protein by Western blot rather than examining the stability of both the RNA and the translated protein. Thus, we chose the TALEN method for gene disruption using the host-transfer technique.

**Fig. 2.**
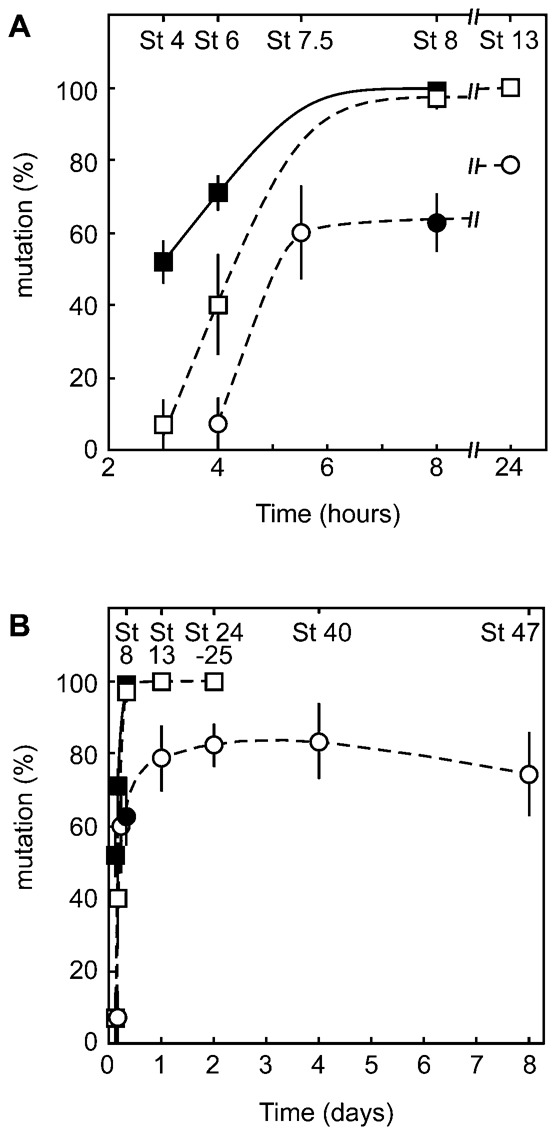
Time course of mutations induced by microinjection with either Tyr-TALEN or Tyr-TALEN-DS mRNAs. Oocytes (squares) or fertilized eggs (circles) were injected with either Tyr-TALEN (open squares and circles) or Tyr-TALEN-DS (closed squares and a circle) mRNAs. The injected oocytes were treated with progesterone, transferred into a host female, and fertilized. The genomic DNA was extracted from four to six individual embryos at the indicated times. The target DNA was amplified by PCR and cloned. The DNA sequence was determined for five to fifteen clones per embryo (supplementary material Figs S1, S2). The data are expressed as the mean value ± standard error. The abscissa is indicated by hours (A) and days (B).

The mutagenic activity of the TALEN-mRNA-injected embryos was assessed by amplification, cloning, and sequence determination of the target gene. The mutation rate of the target sequence in the embryos injected with Tyr-TALEN mRNA at the one -cell stage was 7% at 4 hours post fertilization (hpf) (stage 6), rapidly increased to 60% at 5.5 hpf (stage 7.5), and then slowly increased to 79% up to 24 hpf (stage 13). The mutation rate was maintained at approximately 80% even 8 days after fertilization (Fig. 2; supplementary material Fig. S1). In contrast, the rate in embryos derived from TALEN-mRNA-injected and host-transferred oocytes was 7% at 3 hpf (stage 4), increasing to 40% at 4 hpf (stage 6), and reaching 97% at 8 hpf (stage 8) (Fig. 2; supplementary material Fig. S2). The enhancement of mutagenesis by the host-transfer technique was prominent at 4 hpf.

**Fig. 3.**
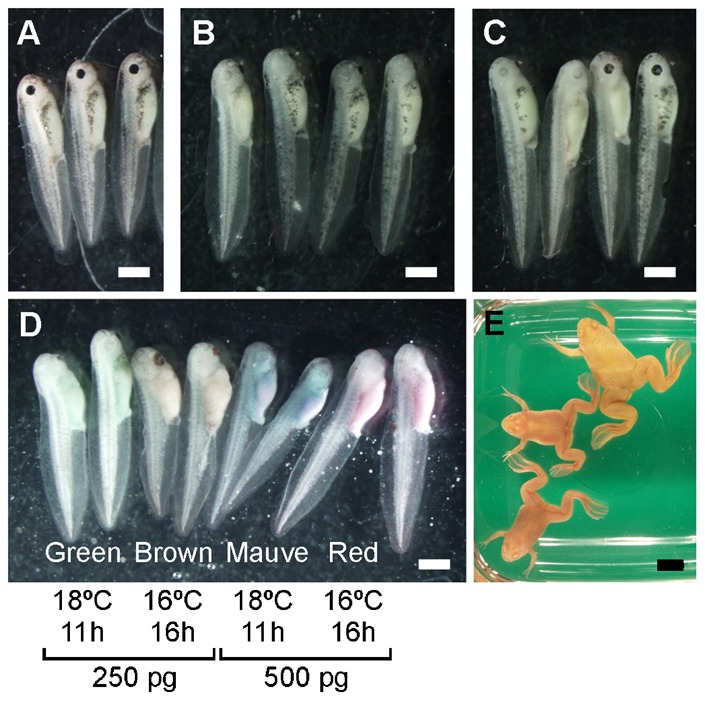
Images of tadpoles and frogs derived from oocytes and embryos injected with Tyr-TALEN mRNAs. (A) Wild-type stage 39–40 tadpoles. (B,C) Fertilized eggs were injected with 500 pg of Tyr-TALEN mRNAs. Four of the stage 39–40 tadpoles were derived from the eggs from two different female frogs (B) and (C). (D) Oocytes were injected with Tyr-TALEN mRNAs, transferred into a host female, and fertilized. Each pair of stage 39–40 tadpoles is colored green, brown, mauve, or red from left to right. The green- and brown-colored tadpoles were derived from oocytes injected with 250 pg of mRNA. The mauve- and red-colored tadpoles were derived from oocytes injected with 500 pg of mRNA. The oocytes were matured by progesterone at 18°C for 11 hours for the green- and mauve-colored tadpoles. The oocytes were matured by progesterone at 16°C for 16 hours for the brown- and red-colored tadpoles. There was no difference between oocytes treated with progesterone at 18°C for 11 hours and those treated at 16°C for 16 hours. Tadpoles were selected randomly. (E) An image of the frogs derived from the oocytes that were injected with Tyr-TALEN mRNAs. Five of seven frogs showed perfect albino phenotypes. Scale bars = 1 mm (A–D) or 1 cm (E).

The Tyr-TALEN-mRNA-injected embryos and mRNA-injected and host-transferred oocytes were allowed to develop to stage 39–40 tadpoles. Loss of pigmentation was more drastic in tadpoles derived from the injected and host-transferred oocytes than in the tadpoles derived from the injected fertilized eggs (Fig. 3). As *X. laevis* is allotetraploid, a nucleus in a somatic cell contains four copies of *tyrosinase* gene. Furthermore, in-frame-mutation has little effect on the gene activity sometimes, depending on the location of the target sequence in the coding region. Only when all four *tyrosinase* genes are inactivated completely in every single cell throughout a whole body, F0 frogs become perfect albinos. Therefore, even if the gene modification rate is 100%, some F0 frogs show partial albino phenotype. Five of seven frogs derived from the injected oocytes were perfect albinos (Fig. 3E).

**Fig. 4.**
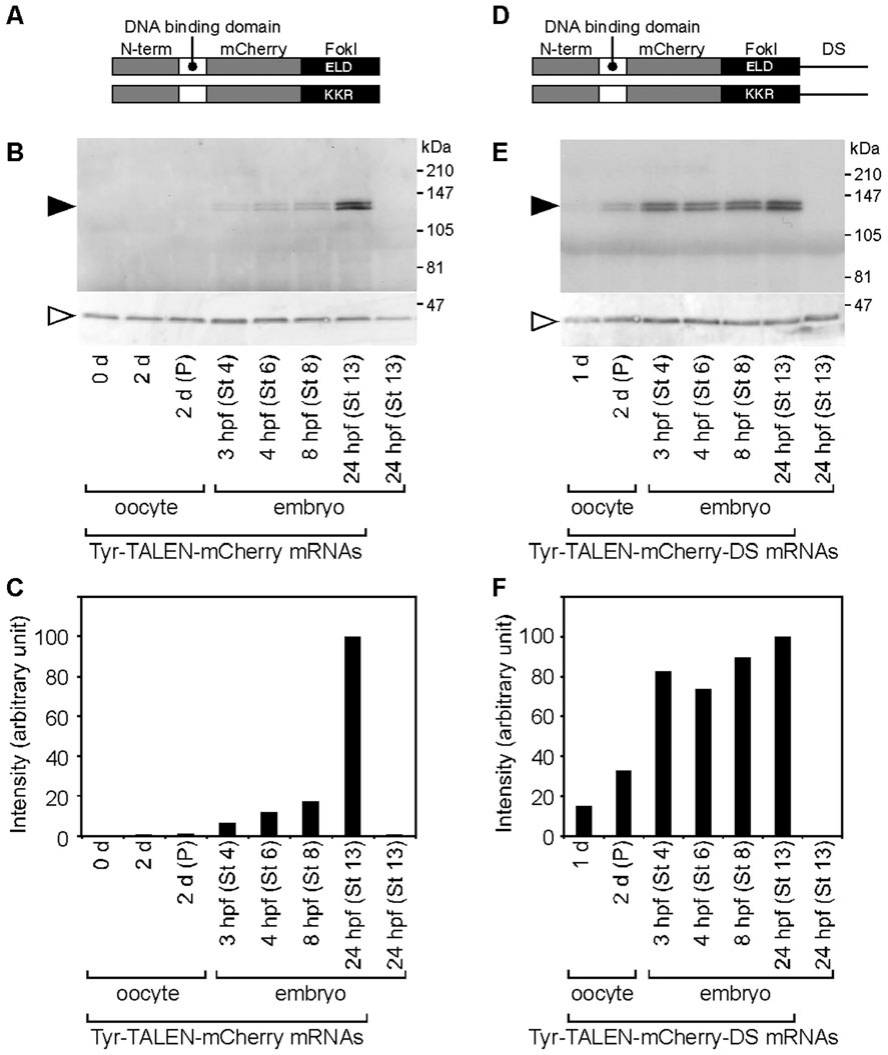
Time course of TALEN-mCherry fusion protein expression in oocytes and embryos. (A,D) Schematic representation of the Tyr-TALEN-mCherry (A) and Tyr-TALEN-mCherry-DS (D) mRNAs. (B,E) Oocytes were injected with either Tyr-TALEN-mCherry (B) or Tyr-TALEN-mCherry-DS (E) mRNAs, transferred into a host female, and fertilized. Five oocytes or host-transferred embryos were homogenized at the indicated time and examined by Western blot analysis using anti-DsRed and anti-actin antibodies. The closed and open arrowheads indicate the positions of the Tyr-TALEN-mCherry and actin proteins, respectively. The stage 13 embryos in the right-most lane were not injected with mRNAs. (C,F) Quantification of the Tyr-TALEN-mCherry protein signals shown in (B,E). The Tyr-TALEN-mCherry proteins were normalized to actin and the signal for the lysates of the stage 13-injected embryos was set to 100%. P, progesterone-treatment.

However, the mutation rate was much lower at 3 hpf in embryos derived from injected and host-transferred oocytes than was expected, as we expected that TALEN proteins would be produced at sufficient levels to cut the target site from two days after injection until fertilization. Tyr-TALEN proteins could not be detected in Western blot analysis using anti-Flag monoclonal antibody. To check the levels of TALEN proteins in the embryos, the C-terminal domain was exchanged for mCherry fluorescent protein between the target DNA-binding domain and the FokI nuclease domain in the TALEN scaffold to generate Tyr-TALEN-mCherry constructs encoding the Tyr-TALEN-mCherry fusion proteins (Fig. 4A). The Tyr-TALEN-mCherry mRNAs were synthesized, injected into oocytes, transferred into a host female, and fertilized. The oocyte or embryo lysates were examined by Western blotting, and the Tyr-TALEN-mCherry fusion proteins were visualized using an anti-DsRed polyclonal antibody (Fig. 4B,C). The fusion proteins were undetectable before fertilization, scarcely detected from 3 to 8 hpf, and expressed at a high level at 24 hpf, indicating that the injected mRNAs were not efficiently translated in the oocytes. These data suggest that the low mutation rate at 3 hpf resulted from a low level of TALEN proteins in the embryos derived from the injected and host-transferred oocytes.

The 3′UTR is involved in the regulation of mRNA translation (Mendez and Richter, 2001). Thus, we examined the translatability in oocytes conferred by fusion of TALEN mRNA with the 3′UTR of maternal mRNAs, such as *Xenopus DEADSouth* and *type-5 actin*. The *DEADSouth* gene encodes an RNA helicase that is translated during mid-oogenesis (King et al., 2005; MacArthur et al., 2000). The mRNA and protein for *type-5 actin* have been observed in the ovary and oocytes, respectively (Mohun and Garrett, 1987). The 3′UTRs of *DEADSouth* and *type-5 actin* were inserted into the Tyr-TALEN-mCherry constructs downstream of the coding region to obtain the Tyr-TALEN-mCherry-DS (Fig. 4D) and -actin mRNAs, respectively. These mRNAs were injected into oocytes, transferred into hosts, and fertilized. Western blot analysis revealed that the TALEN-mCherry fusion proteins were significantly expressed in oocytes two days after Tyr-TALEN-mCherry-DS-mRNA injection. The expression level was similar at 3 and 24 hpf (Fig. 4E,F). The Tyr-TALEN-mCherry-actin mRNAs were inefficiently translated in oocytes compared with the Tyr-TALEN-mCherry-DS mRNAs (data not shown).

The Tyr-TALEN-DS constructs were prepared and transcribed to obtain Tyr-TALEN-DS mRNAs. For embryos derived from oocytes injected with the Tyr-TALEN-DS mRNAs and subjected to the host-transfer technique, the mutation rate was already 52% at 3 hpf, further increasing to 71% at 4 hpf and reaching 99% at 8 hpf, demonstrating that the fusion of TALEN mRNAs with the 3′UTR of *DEADSouth* promoted translation of TALEN mRNA in oocytes and mutagenesis in early embryos.

Highly efficient mutagenesis was observed at 3 hpf in embryos derived from the Tyr-TALEN-DS-mRNA-injected and host-transferred oocytes. Thus, we examined whether the target DNA was modified in the oocytes. No mutations were detected, even six days after oocyte injection with the Tyr-TALEN-DS mRNAs (Table 1).

**Table 1.**
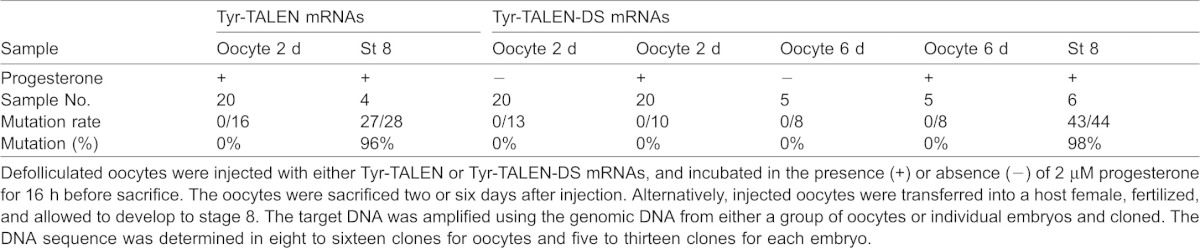
No mutations were induced by TALEN in mature oocytes

## DISCUSSION

We demonstrated that the mutagenic activity of TALENs is enhanced by the host-transfer technique and further improved by injecting TALEN mRNAs fused to the 3′UTR of the *DEADSouth* gene into oocytes. There was approximately a one hour difference between the mutation rate graph of the Tyr-TALEN-mRNA-injected oocytes and that of the injected embryos, implying that one hour is required to prepare the translation complex after mRNA injection. When fertilized eggs were injected with the Tyr-TALEN-DS mRNAs, the mutation rate at 8 hpf (63%) was similar to the estimated rate from embryos injected with Tyr-TALEN mRNAs (Fig. 2A), meaning that there was no difference between the mutation rates in embryos injected with Tyr-TALEN mRNAs and those injected with Tyr-TALEN-DS mRNAs. This observation suggests that the addition of the 3′UTR of the *DEADSouth* gene contributes to TALEN mutagenic activity by promoting TALEN translation in the oocyte to increase TALEN protein levels in early embryos for the more efficient modification of the target gene.

*X. laevis* is an allotetraploid species that resulted from whole-genome duplication after the interspecific hybridization of the diploid species. Because we constructed the Tyr-TALEN DNA binding domains that recognize both *tyrosinase* homoeologs, we generated mutations in almost all of the examined *tyrosinase* genes of randomly selected embryos using our method, which suggests that all four copies of a target gene can be modified in most cells of F0 frogs.

The mutation rate increased in embryos injected with Tyr-TALEN mRNAs by 53% from 4 hpf to 5.5 hpf and by 19% from 5.5 hpf to 24 hpf, after which time it increased minimally, although levels of Tyr-TALEN-mCherry proteins did not decrease till 4 days post fertilization (dpf) (Figs 2 and 4; supplementary material Fig. S3). One explanation for the reduced activity of TALEN in the post-MBT stages is that TALEN mRNAs and proteins containing nuclear localization signals are diluted such that the proliferating target DNA cannot be digested several hours after fertilization. It is known that the levels of some nuclear proteins become too low to maintain physiological function as the cell number and nuclear-to-cytoplasmic ratio increase, especially after the midblastula transition (MBT) around stage 8.5 (9 hpf). For example, the titration of four replication factors during *Xenopus* MBT is essential for slowing the cell cycle and triggering zygotic transcription (Collart et al., 2013). Another explanation may be lack of accessibility to the target DNA. The genome is organized into chromatin that promotes rapid DNA replication in the presence of the large pool of maternal histones. Approaching MBT, the titration of maternal histones and the zygotic expression of transcriptional factors change the state of chromatin assembly in favor of stable transcription (Almouzni and Wolffe, 1995; Prioleau et al., 1994). It is possible that TALEN proteins gain access to the chromatin structure containing maternal histones more easily, where they bind and cut the target DNA.

The stage VI oocytes used for mRNA injection are arrested at prophase in meiosis I and contain condensed chromatins. Progesterone treatment induces germinal vesicle breakdown and chromosome condensation. Meiosis proceeds to the metaphase of meiosis II and stops (Masui and Clarke, 1979). During this transition, nuclear membranes do not reform and chromosomes remain condensed (Furuno et al., 1994). Target DNA modification did not occur in oocytes injected with Tyr-TALEN or Tyr-TALEN-DS mRNAs irrespective of progesterone treatment (the absence of nuclear membrane), even six days after injection. The condensed chromatin structure may block the approach of TALEN to the target site.

Our method combining the oocyte injection of TALEN mRNAs fused to the 3′UTR of the *DEADSouth* gene with host transfer dramatically enhances the mutation rate. Our study shows that gene modification activity can be augmented by increasing the levels of TALEN proteins during early embryogenesis before the MBT.

## Supplementary Material

Supplementary Material
