# Supplementary material for: Highly efficient gene knockout by injection of TALEN mRNAs into oocytes and host transfer in Xenopus laevis
Source: Biol Open. 2015 Jan 16;4(2):180–5. doi: 10.1242/bio.201410009 (PMC4365486; doi:10.1242/bio.201410009)
Supplement: Supplementary Material [file supp_4_2_180__index.html]

Highly efficient gene knockout by injection of TALEN mRNAs into oocytes and host transfer in Xenopus laevis — Highly efficient gene knockout by injection of TALEN mRNAs into oocytes and host transfer in Xenopus laevis — Supplementary Material 

# Highly efficient gene knockout by injection of TALEN mRNAs into oocytes and host transfer in *Xenopus laevis*

## bio.201510009 Supplementary Material

**Files in this Data Supplement:**

- Supplementary Material - Keisuke Nakajima and Yoshio Yaoita doi: 10.1242/bio.201410009
