# Supplementary material for: Highly efficient gene knockout by injection of TALEN mRNAs into oocytes and host transfer in Xenopus laevis
Source: Biol Open. 2015 Jan 16;4(2):180–5. doi: 10.1242/bio.201410009 (PMC4365486; doi:10.1242/bio.201410009)
Supplement: Supplementary Material [file supp_bio.201410009_bio.201510009-s1.pdf]

Biology Open (2015) 000, 1–6 doi:10.1242/bio.201410009

Supplementary Material

Keisuke Nakajima and Yoshio Yaoita doi: 10.1242/bio.201410009

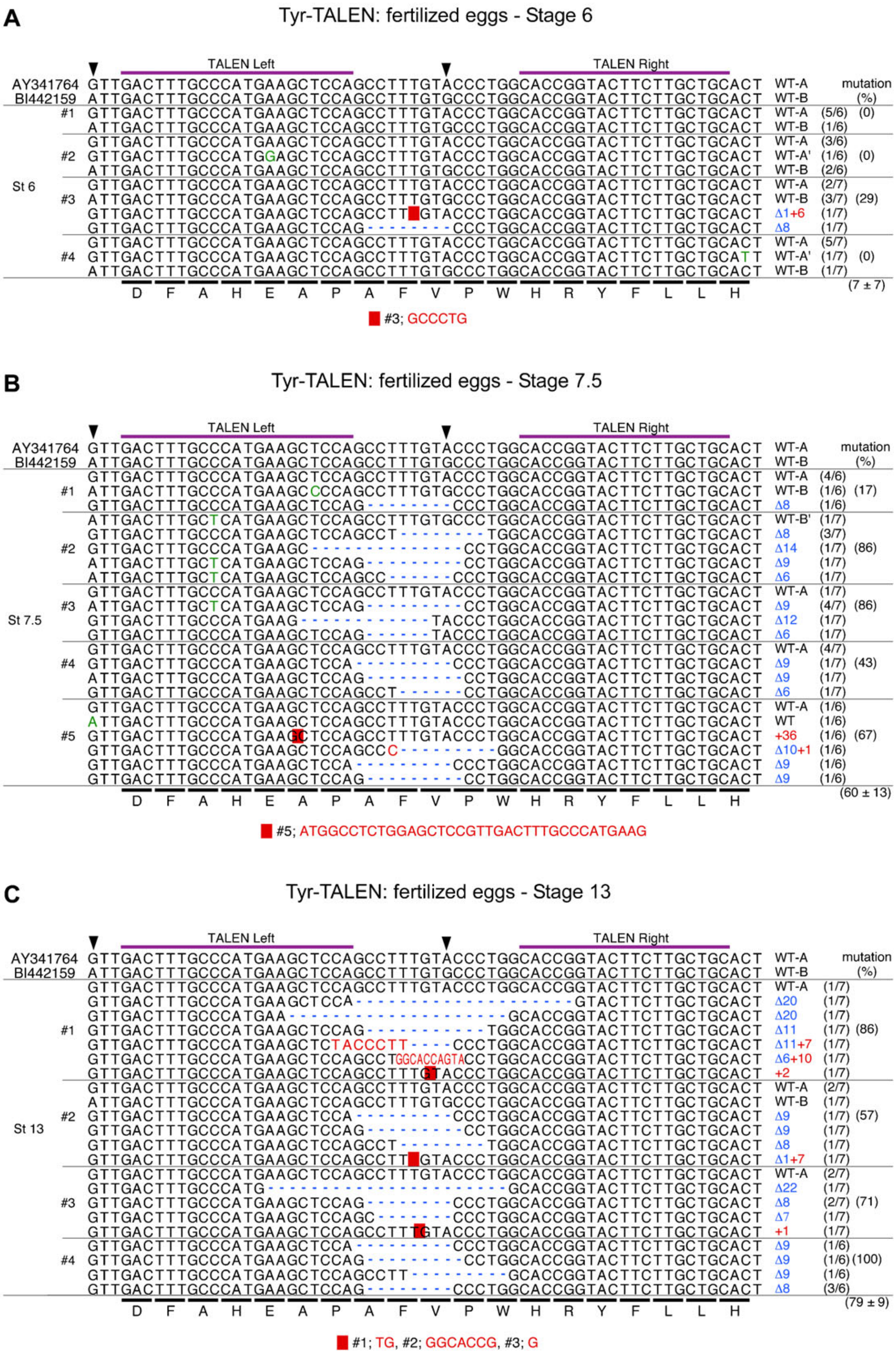

Fig. S1. Analysis of mutations induced by microinjection with Tyr-TALEN or Tyr-TALEN-DS mRNAs into fertilized eggs. (A–G) Genotype analysis of embryos derived from fertilized eggs injected with Tyr-TALEN (A–F) or Tyr-TALEN-DS (G) mRNAs. The target DNA was amplified from the genomic DNA purified from the embryos of the indicated stage and was recloned for sequence determination. The wild-type target DNA and amino acid sequences are indicated on the top and bottom of the panels, respectively. The pair of purple bars represents TALEN binding sites. # refers to a specific embryo. The gaps resulting from deletion (Δ), inserted nucleotides (+), and exchanged nucleotides are denoted as blue dashes, red characters (red squares) and green characters, respectively. The mutation types and frequencies are indicated on the right. Arrowheads indicate the different nucleotide sequences between two *tyrosinase* gene sequences, the accession numbers AY341764 and BI442159.

continued

Biology Open (2015) 000, 1–6 doi:10.1242/bio.201410009

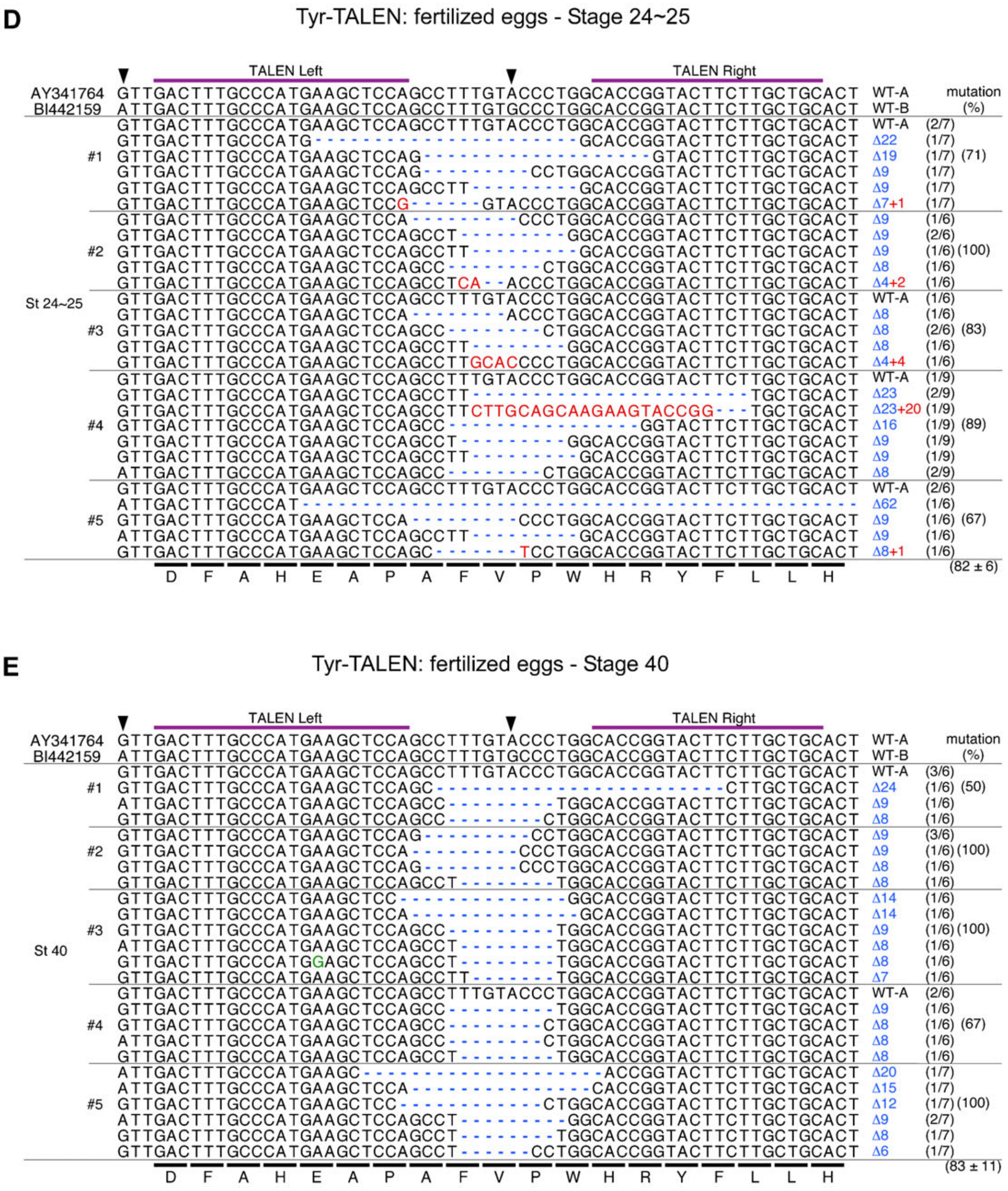

Fig. S1. continued

Biology Open

Biology Open (2015) 000, 1–6 doi:10.1242/bio.201410009

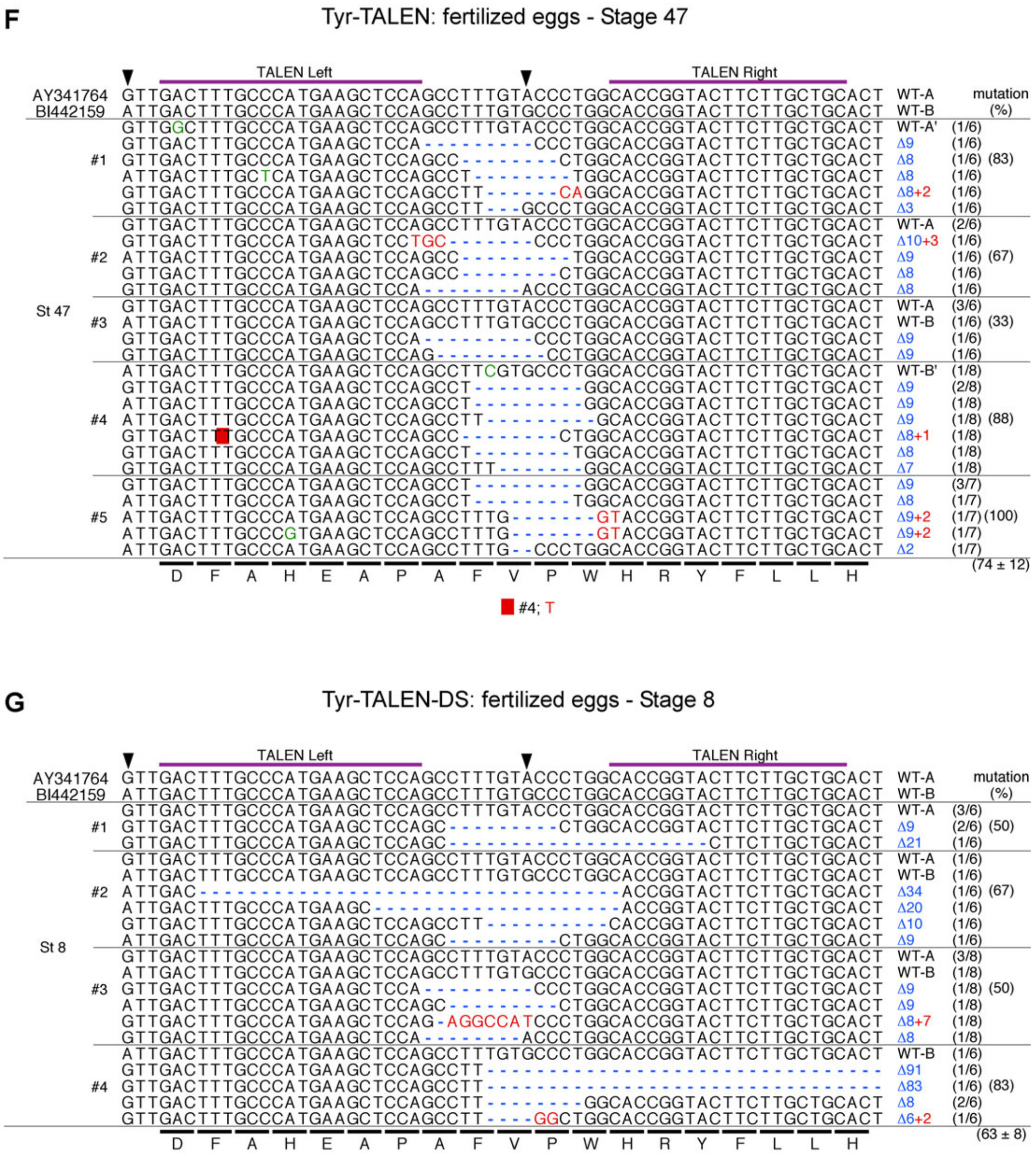

Fig. S1. continued

Biology Open

Biology Open (2015) 000, 1–6 doi:10.1242/bio.201410009

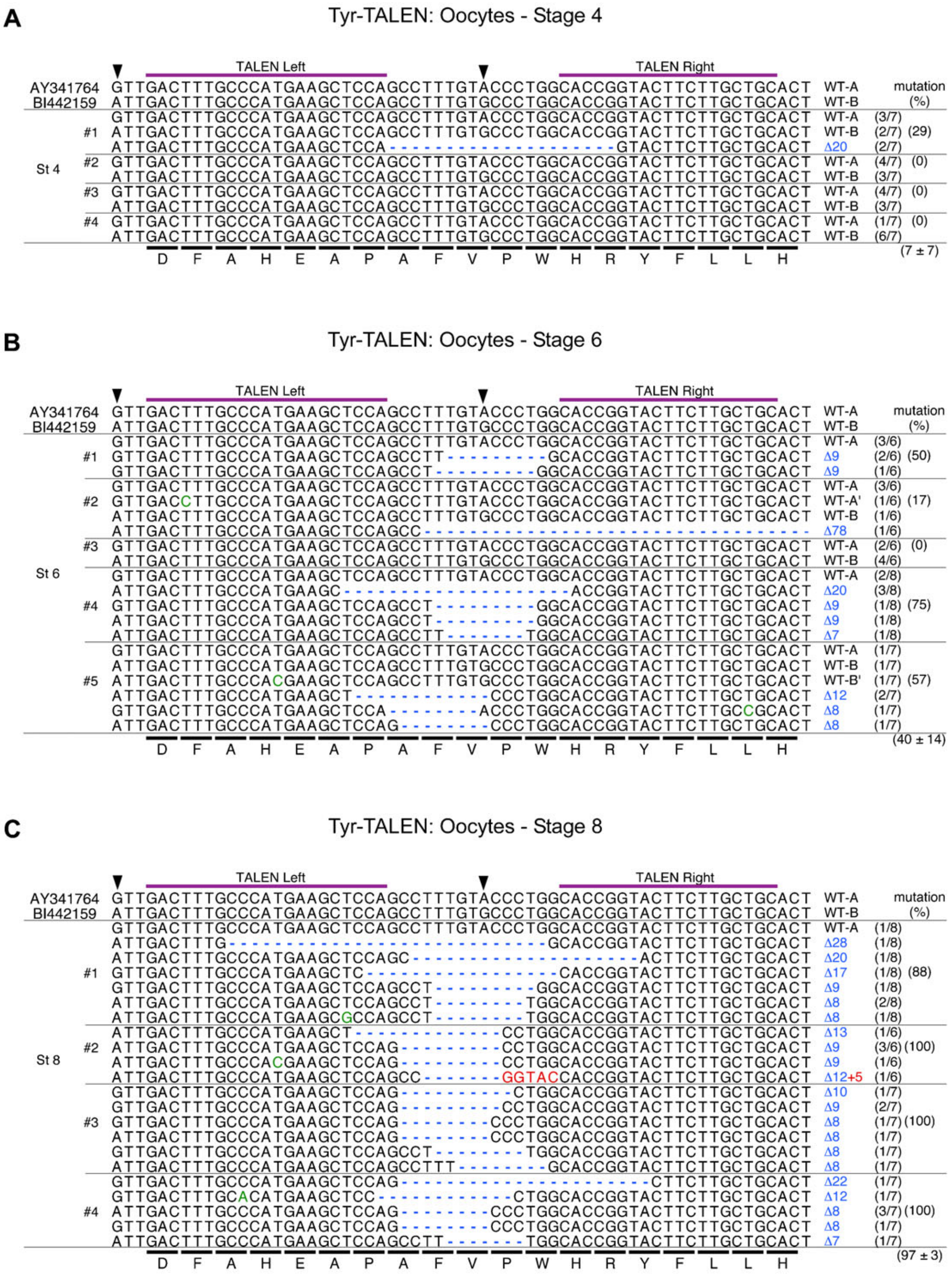

Fig. S2. Analysis of mutations induced by microinjection with Tyr-TALEN or Tyr-TALEN-DS mRNAs into oocytes. (A–H) Genotype analysis of embryos derived from oocytes injected with Tyr-TALEN (A–E) or Tyr-TALEN-DS (F–H) mRNAs. The target DNA was amplified from the genomic DNA purified from the embryos of the indicated stage and was recloned for sequence determination. The alignment is labeled as described in the legend of supplementary material Fig. S1.

continued

Biology Open

Biology Open (2015) 000, 1–6 doi:10.1242/bio.201410009

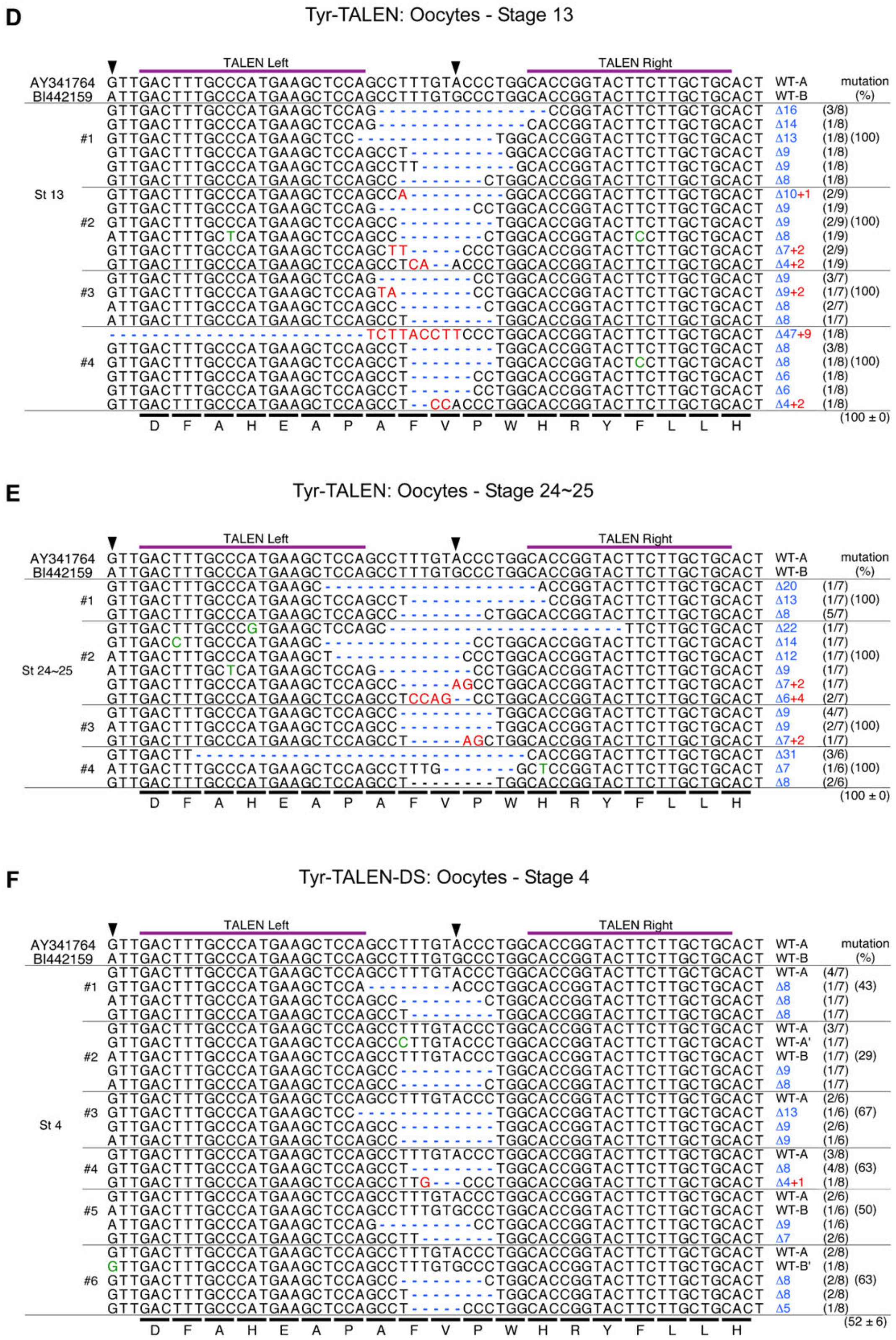

Fig. S2. continued

Biology Open

Biology Open (2015) 000, 1–6 doi:10.1242/bio.201410009

G

Tyr-TALEN-DS: Oocytes - Stage 6

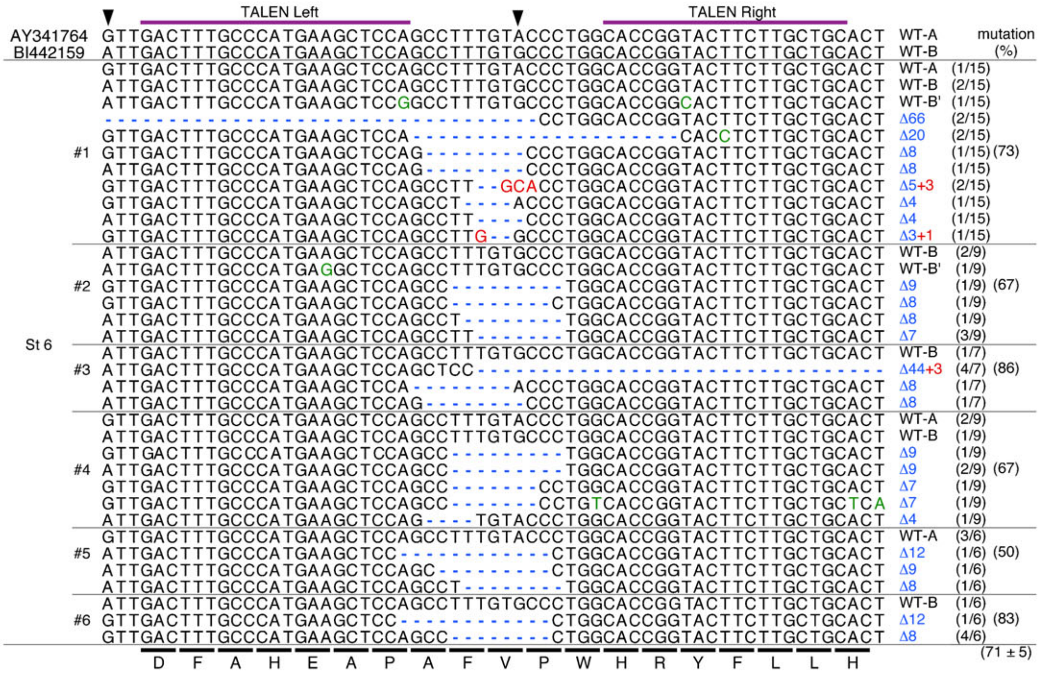

H

Tyr-TALEN-DS: Oocytes - Stage 8

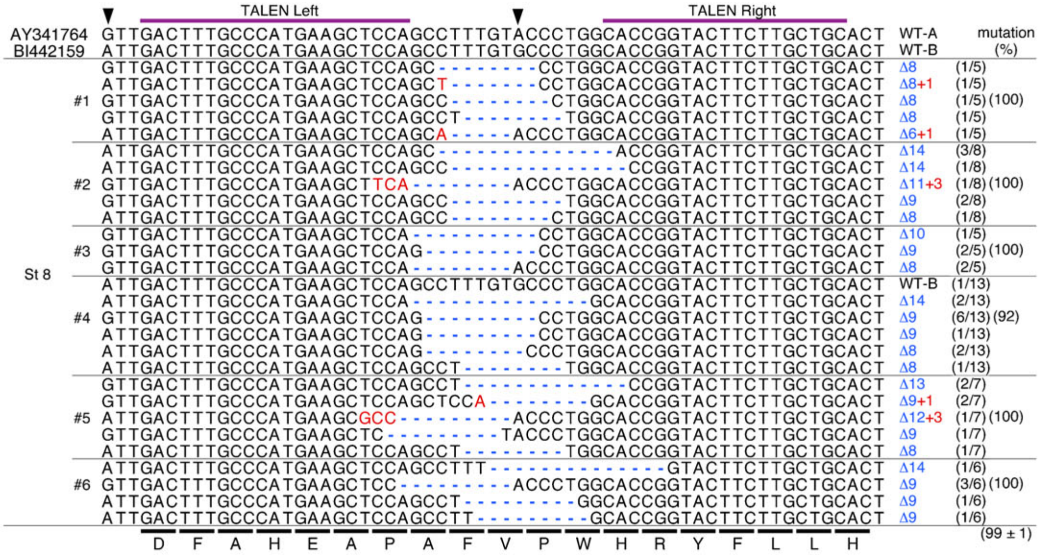

Fig. S2. continued

Biology Open

Biology Open (2015) 000, 1–6 doi:10.1242/bio.201410009

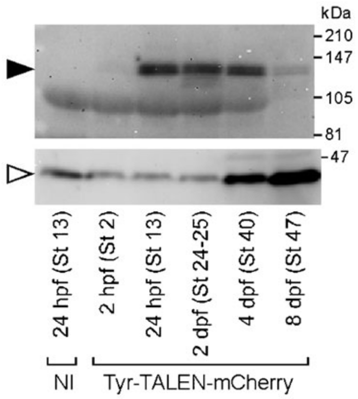

Fig. S3. Time course of TALEN-mCherry fusion protein expression in embryos and tadpoles. Fertilized eggs were injected with Tyr-TALEN-mCherry mRNAs. Five embryos or tadpoles were homogenized at the indicated time and examined by Western blot analysis using anti-DsRed and anti-actin antibodies. The closed and open arrowheads indicate the positions of the Tyr-TALEN-mCherry and actin proteins, respectively. The stage 13 embryos (NI) in the left-most lane were not injected with mRNAs. Note that levels of TALEN-mCherry fusion proteins were not reduced till 4 dpf. A level of actin protein increased at 4 and 8 dpf of tadpoles, which may be ascribed to the development of muscle tissue.

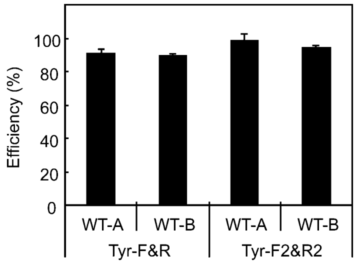

Fig. S4. Amplification efficiency of two homoeologs of *tyrosinase* genes during PCR. Two types of *tyrosinase* genes, WT-A and WT-B, were cloned. The amplification efficiencies using primer sets, Tyr-F and -R and Tyr-F2 and -R2, were determined by Thermal Cycler Dice Real Time System TP850 (TaKaRa). The data are expressed as the mean value ± standard error (n=3).
